# Assessment of Electronic Health Record Search Patterns and Practices by Practitioners in a Large Integrated Health Care System

**DOI:** 10.1001/jamanetworkopen.2020.0512

**Published:** 2020-03-06

**Authors:** Halley Ruppel, Aashish Bhardwaj, Raj N. Manickam, Julia Adler-Milstein, Marc Flagg, Manuel Ballesca, Vincent X. Liu

**Affiliations:** 1 Division of Research, Kaiser Permanente Northern California, Oakland; 2 Department of Medicine, University of California, San Francisco; 3 The Permanente Medical Group, Oakland, California

## Abstract

**Importance:**

The electronic health record (EHR) is a source of practitioner dissatisfaction in part because of challenges with information retrieval. To improve data accessibility, a better understanding of practitioners’ information needs within individual patient records is needed.

**Objective:**

To assess EHR users’ searches using data from a large integrated health care system.

**Design, Setting, and Participants:**

This retrospective cross-sectional analysis used EHR search data from Kaiser Permanente Northern California, an integrated health care delivery system with more than 4.4 million members. Users’ EHR search activity data were obtained from April 1, 2018, to May 15, 2019.

**Main Outcomes and Measures:**

Search term frequency was grouped by user and practitioner types. Network analyses were performed of co-occurring search terms within a single search episode, and centrality measures for search terms (degree and betweenness centrality) were calculated.

**Results:**

A total of 12 313 047 search activities (including 4 328 330 searches and 7 984 717 result views) conducted by 34 735 unique users within 977 160 unique patient EHRs were identified. In aggregate, users searched for 208 374 unique search terms and conducted a median of 4 searches (interquartile range, 1-28 searches). Of all 97 367 active EHR users, 34 735 (35.7%) conducted at least 1 search. However, of all 12 968 active EHR physician users, 9801 (75.6%) conducted at least 1 search, and of all 1908 active pharmacist users, 1402 (73.5%) conducted at least 1 search. The top 3 most commonly searched terms were *statin* (75 017 searches [1.7%]), *colonoscopy* (73 545 [1.7%]), and *pft* (54 990 [1.3%]). However, wide variation in top searches were noted across practitioner groups. Terms searched most often with another term in a single linked search episode included *statin*, *lisinopril*, *colonoscopy*, *gabapentin*, and *aspirin*.

**Conclusions and Relevance:**

Although physicians and pharmacists were the most active users of EHR searches, search volume and frequently searched terms varied considerably by and within user role. Further customization of the EHR interface may help leverage users’ search content and patterns to improve targeted information retrieval.

## Key Points

**Question:**

What are the electronic health record search patterns and practices among electronic health record users?

**Findings:**

In this cross-sectional study of 12 313 047 patient electronic health record search activities, physicians and pharmacists conducted the most searches, with *statin*, *colonoscopy,* and *pft* being the most frequently searched terms. Search term networks revealed how users bridged together data across various domains relevant to a single clinical interaction.

**Meaning:**

Our results suggest that opportunities exist for improving the clinical use of the electronic health record by identifying potential areas to customize information retrieval based on diverse user roles and linked search approaches.

## Teaser

This cross-sectional study uses data from a large integrated health care system to assess search patterns and practices among electronic health record users.

## Introduction

The electronic health record (EHR) can be a major source of dissatisfaction for practitioners, contributing to burnout and stress.^1,2^ Practitioners and patients largely agree that practitioners spend too much time in front of the computer, often at the expense of engaging with the patient.^3,4,5,6,7,8^ Two major reasons for the excessive time spent in the EHR system include burdensome documentation demands and the inaccessibility of data.^2,4,9^ Although numerous studies^10,11,12,13,14,15^ have examined documentation burden and practices, the challenges of EHR information retrieval in clinical practice remain poorly understood.^16,17,18,19^ Studying information retrieval is critically important because variability in documentation practices, usability challenges, and propagation of erroneous information^15,20^ result in the need to navigate large volumes of low-value EHR data to locate clinically actionable patient information. Failure to identify relevant clinical data can lead to overuse of treatments or procedures, provision of low-quality care, and occurrence of medical errors.^15,21^

An emerging mechanism used for patient information retrieval is the EHR search feature. Analyzing EHR user search patterns can reveal information needs by showing the types of information that may be difficult to locate through other means. In other applications, search data, such as Google Trends, are often used to study the types of information sought by populations.^22^ Widely available, comprehensive patient record search features are relatively new to the EHR, and their use has not been extensively studied.^16,17,23^ In addition to identifying information needs, examining searches by EHR user type can reveal the overall demand or penetration of the search functionality. Exploring terms frequently searched together can highlight patterns that can then be used to make data gathering in the EHR more intuitive and user-friendly. In this study, we analyzed more than 12 million EHR search activities from a large, highly integrated health care delivery system to assess search behaviors and identify opportunities to improve targeted information retrieval in clinical practice across a diverse set of users.

## Methods

For this cross-sectional study, we evaluated EHR search data from Kaiser Permanente Northern California (KPNC), an integrated health care delivery system including 21 hospitals and serving 4.4 million members. KPNC began deploying a single comprehensive EHR system (Epic) in 2007. The EHR, known internally as KP HealthConnect, is used across all KPNC inpatient and outpatient facilities.^24^ KPNC determined that this study did not meet the definition of human subject research, and the study was therefore exempted from institutional review board approval. Patient and user identifications were blinded before analysis. This study followed the Strengthening the Reporting of Observational Studies in Epidemiology (STROBE) reporting guideline.

Starting in 2015, the Chart Search feature was made available in KP HealthConnect and allows users to search for specific terms within an individual patient’s EHR in structured and unstructured data (eg, clinical notes, laboratory data, medications, imaging, procedures, orders, allergies, immunizations, and problem list fields).^25^ Searches can match on the exact terms entered and synonyms generated through Systematized Nomenclature of Medicine ontologies. Results of the search are displayed in a list format with some contextual information when the search successfully finds a matching term or an indication that no results match the term. Users can click a positive search result, which brings them to the selected location of the term in the patient’s record.

### EHR Search Data

We retrospectively evaluated KPNC EHR audit log data for search activities between April 1, 2018, and May 15, 2019. A search activity includes the search term itself or a view of a search result (click or hover). For each search activity, we retrieved the following information: search term; time stamp; blinded user and patient identifiers; search session identifier; and, when applicable, licensed practitioner type (eg, physician, nurse, pharmacist, or physician trainees) and specialty type (eg, internal medicine). For analysis, we further grouped licensed practitioners by discipline: physicians (including podiatrists and optometrists); nurses (registered nurses and licensed vocational nurses); nonphysician mental health practitioners (eg, social workers, therapists, and psychologists); pharmacists; rehabilitation therapists (eg, occupational therapists, physical therapists, and speech therapists); advanced practice providers (APPs; eg, nurse practitioners and physician assistants); medical assistants; and other. Note that not all EHR users are practitioners licensed to provide clinical care to patients (eg, administrative personnel and researchers).

We used search term data in their recorded format to reflect how users were conducting searches but converted all terms to lowercase and removed nonalphanumeric characters and spaces. We produced summary statistics (eg, median and interquartile range [IQR]) for frequency of searches by user (using a denominator of all active EHR users defined as a user with any audit log activity data), practitioner group and time of day, and result views per search.

To identify the most frequently searched clinical domains, we aggregated search terms using the Observational Health Data Sciences and Informatics (OHDSI) Standard Vocabulary synonym and domain tables.^26^ Because the same search term could match multiple OHDSI domains, we sequentially categorized terms within domains in the following order: condition, procedure, drug, observation, measurement or measured value, and other.

### Linked Search Networks

To better understand search patterns, we examined terms that were searched together within a single search episode in the same patient record. We created search episodes by grouping searches conducted by the same user for the same patient in a single EHR session (based on unique session identification numbers). We used the phrase *linked search terms* to indicate a pair of search terms that occurred within the same search episode. We displayed these linked search terms using network graphs^27^ for the top-searching practitioner groups (physicians, pharmacists, nurses, nonphysician mental health, and APPs) and top-searching physician specialty (internal medicine). To identify highly interconnected terms (terms that appeared in multiple linked search term pairs across users), we calculated weighted degree and betweenness centrality metrics for each included term. Degree centrality describes the number of terms to which that term was linked during search episodes in our data set, whereas betweenness describes how often a node (search term) is in the shortest path between 2 other nodes.

### Statistical Analysis

We used a χ^2^ test to examine the association between practitioner group and use of the search feature. A 2-sided *P* < .05 was considered to be statistically significant. All analyses were conducted in R, version 3.5.2 (The R Project for Statistical Computing), using packages including ggplot2, ggnet, and tidygraph.

## Results

During the 13-month study period, 34 735 unique users performed 12 313 047 search activities, including 4 328 330 searches and 7 984 717 result views within 977 160 unique patient EHRs. In aggregate, users searched for 208 374 unique search terms. For 1 647 643 searches (38.1%), no results were viewed (click or hover), and the median number of results viewed per search was 1 (IQR, 0-3 result views).

Table 1 displays the overall search characteristics. Users of the search feature conducted a median of 4 searches (IQR, 1-28 searches). The top 5% of search feature users searched more than 584 times during the study period, with a maximum of 51 784 searches by a single user. Grouped by time of day, most searches (2 178 074 [50.3%]) were conducted between noon and 6 pm. In eFigure 1 in the Supplement, we present search frequency by hour of the day for each practitioner group.

**Table 1.** Characteristics of Searches Performed From April 1, 2018, to May 15, 2019

Characteristic	Searches^a^ (N = 4 328 330)
Searches per user of search feature	
Median (IQR)	4 (1-28)
Range	1-51 784
Time of day	
Morning (6:00-11:59 am)	1 794 822 (41.5)
Afternoon (12:00-5:59 pm)	2 178 074 (50.3)
Evening (6:00-11:59 pm)	296 306 (6.8)
Night (12:00-5:59 am)	59 128 (1.4)
Practitioner groups with most searches^b^	
Physician	2 042 804 (47.2)
Pharmacist	689 852 (15.9)
Nurse	239 379 (5.5)
Mental health practitioner	120 559 (2.8)
APP	116 763 (2.7)

Abbreviations: APP, advanced practice provider; IQR, interquartile range.

^a^ Data are presented as number (percentage) of searches unless otherwise indicated.

^b^ Physician includes practitioners with MD, DO, OD, and DPM degrees and residents. Nurse includes registered nurses, licensed vocational nurses, and licensed practical nurses. Mental health practitioner includes nonphysician practitioners, such as social workers, therapists, and psychologists. APP includes advanced practice nurses and physician assistants.

### Search Characteristics by Practitioner Groups

The 5 practitioner groups conducting the most searches were physicians (2 042 804 [47.2%]), followed by pharmacists (689 852 [15.9%]) and nurses (239 379 [5.5%]). Of all 97 367 EHR users with any audit log activity data, 34 735 (35.7%) conducted at least 1 search. However, among 12 968 physicians, 9801 (75.6%) conducted at least 1 search, and among 1908 pharmacists, 1402 (73.5%) conducted at least 1 search (Table 2). The χ^2^ test demonstrated a significant association between clinician group and use of the search feature (χ^2^_4_, 7357.6; n = 24 587; *P* < .001). Those who conducted a search were more likely to be a physician or pharmacist than those who did not conduct a search.

**Table 2.** Users Conducting Searches per Practitioner Group

Practitioner Group^a^	Unique Users of EHR, No.^b^	Unique Users Conducting a Search, No. (%)
Nurses	27 548	8848 (32.1)
Physicians	12 968	9801 (75.6)
Mental health practitioners	3113	1590 (51.1)
Pharmacists	1908	1402 (73.5)
APPs	1760	1069 (60.7)

Abbreviations: APPs, advanced practice providers; EHR, electronic health record.

^a^ Physician includes practitioners with MD, DO, OD, and DPM degrees and residents. Nurses include registered nurses, licensed vocational nurses, and licensed practical nurses. Mental health practitioners include nonphysician practitioners, such as social workers, therapists, and psychologists. APPs include advanced practice nurses and physician assistants.

^b^ EHR users with any audit log activity.

Of those conducting at least 1 search, physicians conducted a median of 30 searches each (IQR, 5-179 searches), pharmacists conducted a median of 76 searches (IQR, 5-587 searches), and nurses conducted a median of 2 searches (IQR, 1-4 searches). Among searches by physicians, the specialties conducting the most searches were internal medicine (931 067 [45.6%]), family practice (226 472 [11.1%]), and psychiatry (130 317 [6.4%]).

### Most Frequently Searched Terms and Domains

Table 3 displays the most frequently searched terms overall; *statin* (75 017 searches [1.7%]), *colonoscopy* (73 545 [1.7%]), and *pft* (54 990 [1.3%]) accounted for 4.7% of all searches. Overall, *statin* was searched by 2018 users (5.8% of users who conducted a search), *colonoscopy* was searched by 4130 (11.9%), and *pft* was searched by 2561 (7.4%). However, the most frequently searched terms differed substantially by practitioner groups, and no single term appeared in the top 10 most frequent searches of more than 2 practitioner groups (Table 4). For example, physicians most commonly searched for *colonoscopy*, *pft*, and *ppd,* whereas pharmacists most frequently searched medications, including *statin*, *metformin*, and *lisinopril*. When grouped by OHDSI domains, physicians most frequently searched for drugs and conditions (eg, *sleep apnea* and *asthma*), pharmacists most frequently searched for drugs, and nurses searched for observations (eg, *A1c* and *mood*) and conditions (eFigure 2 in the Supplement).

**Table 3.** Overall Top 20 Most Frequently Searched Terms From April 1, 2018, to May 15, 2019^a^

Rating	Search Term	No. (%) of Searches (N = 4 328 330)
1	*statin*	75 017 (1.7)
2	*colonoscopy*	73 545 (1.7)
3	*pft*	54 990 (1.3)
4	*palliative care*	34 258 (0.8)
5	*asthma*	28 277 (0.7)
6	*metformin*	27 159 (0.6)
7	*depression*	24 469 (0.6)
8	*lisinopril*	23 976 (0.6)
9	*ppd*	23 514 (0.5)
10	*gabapentin*	22 578 (0.5)
11	*copd*	21 017 (0.5)
12	*phq*	20 450 (0.5)
13	*sleep apnea*	18 534 (0.4)
14	*moca*	18 086 (0.4)
15	*palliative*	17 472 (0.4)
16	*cough*	15 657 (0.4)
17	*aspirin*	15 283 (0.4)
18	*sleep study*	14 452 (0.3)
19	*atorvastatin*	13 878 (0.3)
20	*prednisone*	13 327 (0.3)

^a^ Capitalization of search terms standardized and nonalphanumeric characters removed.

**Table 4.** Top 10 Most Frequently Searched Terms, by Top-Searching Practitioner Groups^a^

Term	No. (%) of Searches
**Physician Searches (n = 2 042 804)**	
*colonoscopy*	61 166 (3.0)
*pft*	21 135 (1.0)
*ppd*	19 179 (0.9)
*sleep apnea*	15 354 (0.8)
*gabapentin*	15 113 (0.7)
*sleep study*	12 265 (0.6)
*egd*	11 050 (0.5)
*prednisone*	10 977 (0.5)
*asthma*	10 730 (0.5)
*tsh*	10 076 (0.5)
**Pharmacist Searches (n = 689 852)**	
*statin*	52 708 (7.6)
*metformin*	20 630 (3.0)
*lisinopril*	14 857 (2.2)
*glipizide*	10 185 (1.5)
*aspirin*	9500 (1.4)
*atorvastatin*	9396 (1.4)
*losartan*	8653 (1.3)
*amlodipine*	6553 (0.9)
*gabapentin*	5975 (0.9)
*advair*	5920 (0.9)
**Nurse Searches (n = 239 379)**	
*palliative care*	11 393 (4.8)
*palliative*	6320 (2.6)
*statin*	6060 (2.5)
*foley*	5843 (2.4)
*cycle order*	4757 (2.0)
*hbsag*	4266 (1.8)
*aneurysm*	2820 (1.2)
*conditional*	2552 (1.1)
*mammo*	2207 (0.9)
*pft*	2006 (0.8)
**Mental Health Practitioner Searches (n = 120 559)**	
*social work*	10 673 (8.9)
*social services*	7611 (6.3)
*msw*	6286 (5.2)
*palliative care*	4868 (4.0)
*palliative*	3792 (3.1)
*social worker*	3619 (3.0)
*memory*	3078 (2.6)
*moca*	2752 (2.3)
*spd*	1883 (1.6)
*social*	1493 (1.2)
**APP Searches (n = 116 763)**	
*op note*	4712 (4.0)
*moca*	2778 (2.4)
*colonoscopy*	2696 (2.3)
*ahi*	2258 (1.9)
*memory*	2187 (1.9)
*skin cancer*	1993 (1.7)
*preop*	1923 (1.6)
*mmse*	1590 (1.4)
*fibroscan*	1211 (1.0)
*sleep apnea*	1183 (1.0)

Abbreviation: APP, advanced practice provider.

^a^ Capitalization of search terms standardized and nonalphanumeric characters removed. Physician includes practitioners with MD, DO, OD, and DPM degrees and residents. Nurse includes registered nurses, licensed vocational nurses, and licensed practical nurses. Mental health practitioner includes nonphysician practitioners, such as social workers, therapists, and psychologists. APP includes advanced practice nurses and physician assistants.

### Linked Searches Within Episodes

The 4 328 330 searches in this study were conducted within 2 502 802 unique episodes (a single EHR session by a single user for the same patient). The Figure displays the network graph of linked search terms for the top 5 practitioner groups (physicians, pharmacists, nurses, mental health practitioners, and APPs). Dense search networks of linked search terms clustered around clinical topics, including cardiometabolic disease, mental health, routine diagnostic testing, respiratory disease, cognitive testing, and end-of-life care. For example, APP searches showed networks for procedures and diagnostic workups (eg, *stress test*, *catheterization*, and *echo*). Linked searches conducted by nonphysician mental health practitioners included pairs of terms such as *social work* and *social services*. Pharmacists commonly searched medications (eg, *lisinopril*, *statin*, and *aspirin*) in tandem with specific conditions (eg, *heart attack*, *angina*, and *palpitation*). Pharmacists also searched for changes in drug regimens using terms such as *discontinue* or *decrease* with medication names. eFigure 3 in the Supplement presents a focused network graph for internal medicine physicians (the specialty conducting the most searches).

**Figure.**
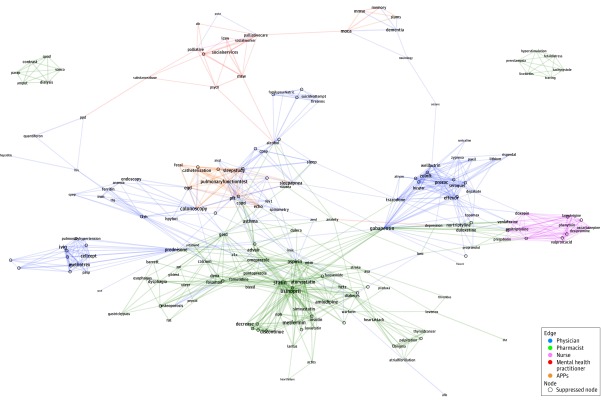
Network Graph of the Top 2% of Linked Searches Performed by the Top 5 Practitioner Groups Edge colors were assigned to the single practitioner group with the highest frequency of searches for the pair of terms; however, the search may have been prevalent in other practitioner groups as well. For visual clarity, the network was restricted to the top 2% of the most frequently searched pairs of terms, which represent 0.6% of the 208 374 unique search terms. In addition, terms with fewer than 4 other terms connected to it were suppressed in this graph. The size of the search term text corresponds to the frequency with which that term was searched. APPs indicates advanced practice providers.

### Search Terms by Centrality

The most highly interconnected terms are listed in the eTable in the Supplement. Those terms with the highest degree centrality included *statin*, *lisinopril*, *colonoscopy*, *gabapentin*, and *aspirin*. Key terms bridging distinct clusters, based on betweenness centrality, were *aspirin*, *statin*, *colonoscopy*, *alcohol*, and *gabapentin*. For example, *alcohol* bridged search concepts related to screening practices, suicide, and social services.

## Discussion

Within slightly more than 1 year of data from a large, integrated health care delivery system, more than 12 million EHR search activities were conducted by more than 34 000 users for nearly 1 million patients. We found that the EHR search feature was used by nearly three-quarters of physicians and pharmacists, but the median number of searches per search feature user was low. The high degree of variability in top search terms across practitioner groups underscores the wide diversity of information needs. Our evaluation of search networks also situated individual terms in their clinical context and revealed terms with high degrees of interconnectedness. Together, these results suggest that search activity data may be used to inform efforts to facilitate EHR information retrieval.

Although most physicians and pharmacists used the search feature, only approximately one-third of all active EHR users conducted any search. Among those who had conducted a search, there was a median of only 4 searches per user for slightly more than a year. Search feature use was especially low among nurses, who represented the single largest group of practitioners. These findings may indicate that EHR design is better suited to nurses’ information needs; however, other literature does not support this hypothesis.^28^ Instead, we suspect that this finding reflects an opportunity for further penetration of the search feature among users and highly varied workflows among practitioners. For example, the EHR may be the primary source of contextual information for pharmacists who lack direct patient contact. To support practitioners, such as pharmacists, who frequently use the search feature to locate similar information, structured searches that can be customized could improve strategies often reused across patient records.^29^ User-specific customization could also be accomplished by tailoring EHR displays based on the user’s prior searches and information retrieval history.^30^

We found that the most frequently searched terms were also common reasons for receiving health care (eg, *statin*, *colonoscopy,* and *pft*). Although the most frequently searched terms were used in less than 2% of searches, within practitioner groups, the top searches were less sparse. For example, nearly 8% of pharmacists’ searches were for *statin,* and the top 10 search terms by pharmacists composed nearly 20% of all their searches. Identification of highly searched terms provides an empirical method for determining concepts that would benefit from improved navigation pathways in the EHR. Variation in search patterns across practitioner groups suggests that there may be opportunities for further customization of the EHR interface by practitioner type. Customization is an important determinant of EHR satisfaction and has been recognized as a critical consideration in EHR redesign.^31,32^

The contents of the searches in our study also underscore the challenges that practitioners face when attempting to develop a comprehensive clinical picture from siloed information within the EHR (ie, information fragmentation^33,34^). The network graphs showed linked searches across diverse domains (eg, medications, diagnoses, notes, and images) on a single clinical problem. Additional efforts to empirically link relevant items across domains may further improve EHR usability.^35,36^ For example, *aspirin* was searched with terms such as *statin*, *catheterization*, *metformin*, and *pradaxa*, which makes intuitive clinical sense and could be combined within a cardiometabolic disease and treatment pathway. In addition, a substantial portion of linked searches involved mental health concerns (eg, drugs, services, substance use, family history, and cognitive function), suggesting the need for a clustered presentation of mental health–related information.

Although the search feature is a key tool for efficiently retrieving information from the mass of EHR data, we identified several uses of the search feature that seem to be forced inefficiencies^37^ of poor EHR design. For example, pharmacists’ searches for terms such as *decrease* and *discontinue* highlight limitations in their ability to pinpoint medication changes over time. A timeline display of medication orders, administrations, and refills could help pharmacists understand prescribing history more readily. Mental health practitioners commonly searched synonyms for social work (eg, *lcsw*, *msw*, or *social services*) together within a single search episode, suggesting specific challenges finding information on social services. The search feature may also need improved capabilities for retrieving synonyms and abbreviations, as well as related clinical information.^38,39^

The present study contributes to the scant literature on EHR searching, which focuses primarily on homegrown solutions for searching within or across patient records.^16,17,23,40^ One of the only prior studies^16^ of searches within patient records included 2207 EHR searches by 436 unique users at a single institution and found that the most common searches were for laboratory or test results. In the present study, top searches across all practitioner groups were dominated by drugs, procedures, conditions, and clinical services, such as palliative care. This difference may be related to the size and scope of our study and the near-decade of EHR advancements since the publication of the prior study.^16^ A number of studies^18,19,30,41^ have explored practitioners’ information-seeking practices in general and EHR navigation, typically in small samples of practitioners and often using methods such as direct observation.

The present study adds to the current information on EHR searches because of the breadth of the data, which provide a comprehensive overview of frequency and content of searches within patient records. However, we were unable to determine contextual information, such as why searches were conducted and whether users were satisfied with the results. For example, no results were viewed (click or hover) in more than one-third of searches, which might indicate that the search feature fails to meet users’ information needs. Alternatively, the finding may indicate that the search was successful because the specific term never appeared in the patient record or because it did but did not require further examination.

Opportunity exists for future investigations to contextualize these findings using additional data sources and research methods. The EHR audit log data (including tasks performed before and after the search), patient-level data, and practitioner interviews and observations could help explain why a search was conducted and whether the search was successful. For example, practitioners could be interviewed or surveyed about barriers to locating information related to the top search terms identified in this study. Temporal data could be used to examine how search habits change over time as practitioners learn the tool or gain clinical expertise. There is also ample opportunity for deeper exploration of search patterns of specific practitioner subgroups.

### Limitations

This study has limitations. First, we did not attempt to address spelling errors or abbreviations when aggregating search terms. The summary statistics therefore likely underestimate search term frequency. Second, the data represent searches within the Epic Chart Search feature and may not be generalizable to other EHR systems. Third, KPNC members receive most of their care within the KPNC system, and as a result, practitioners’ EHR search practices may not be generalizable. However, given the widespread use of Epic and the increasing presence of integrated health care systems in the United States, the findings may be increasingly relevant.

## Conclusions

In this study, we identified potential opportunities to improve EHR navigation by examining the searches performed by EHR users within patient records. The findings suggest that key areas for improving EHR information retrieval include linking information related to key clinical areas as well as further customization of the EHR interface across user types. Our findings from these large-scale search data appear to offer an empirical approach to improving EHR design based directly on user information needs.

## Notes

Supplement. **eTable.** Search Terms Ranked by Centrality (Degree or Betweenness) for Top 5 Practitioner Groups **eFigure 1.** Search Frequency by Hour of Day for Practitioner Groups **eFigure 2.** Search Frequency by OHDSI Domain Within Practitioner Groups **eFigure 3.** Network Graph: Top 5% of Linked Searches Performed by Physicians in Internal Medicine
