# Supplementary material for: Assessment of Electronic Health Record Search Patterns and Practices by Practitioners in a Large Integrated Health Care System
Source: JAMA Netw Open. 2020 Mar 6;3(3):e200512. doi: 10.1001/jamanetworkopen.2020.0512 (PMC7060491; doi:10.1001/jamanetworkopen.2020.0512)
Supplement: Supplement. — eTable. Search Terms Ranked by Centrality (Degree or Betweenness) for Top 5 Practitioner Groups eFigure 1. Search Frequency by Hour of Day for Practitioner Groups eFigure 2. Search Frequency by OHDSI Domain Within Practitioner Groups eFigure 3. Network Graph: Top 5% of Linked Searches Performed by Physicians in Internal Medicine [file jamanetwopen-3-e200512-s001.pdf]

Supplementary Online Content

Ruppel H, Bhardwaj A, Manickam RN, et al. Assessment of electronic health record search patterns and practices by practitioners in a large integrated health care system. *JAMA Netw Open*. 2020;3(3):e200512. doi:10.1001/jamanetworkopen.2020.0512

eTable. Search Terms Ranked by Centrality (Degree or Betweenness) for Top 5 Practitioner Groups

eFigure 1. Search Frequency by Hour of Day for Practitioner Groups

eFigure 2. Search Frequency by OHDSI Domain Within Practitioner Groups

eFigure 3. Network Graph: Top 5% of Linked Searches Performed by Physicians in Internal Medicine

This supplementary material has been provided by the authors to give readers additional information about their work.

© 2020 Ruppel H et al. *JAMA Network Open.*

eTable 1. Search terms ranked by centrality (degree or betweenness) for top 5 practitioner groups.

Rank	Degree	Betweenness
**1**	*statin*	*aspirin*
**2**	*lisinopril*	*statin*
**3**	*colonoscopy*	*colonoscopy*
**4**	*gabapentin*	*alcohol*
**5**	*aspirin*	*gabapentin*
**6**	*moca*	*trazadone*
**7**	*losartan*	*msw*
**8**	*pft*	*hiv*
**9**	*metformin*	*lisinopril*
**10**	*mmse*	*gerd*

© 2020 Ruppel H et al. *JAMA Network Open.*

eFigure 1. Search frequency by hour of day for practitioner groups.

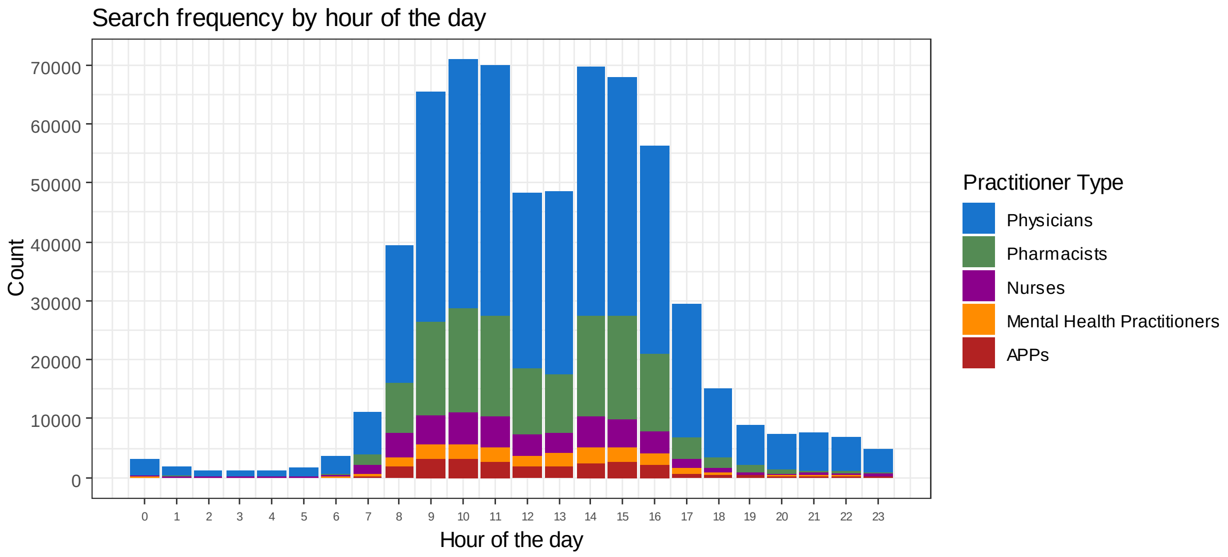

APP= Advanced Practice Provider

© 2020 Ruppel H et al. *JAMA Network Open.*

eFigure 2. Search frequency by OHDSI domain within practitioner groups.

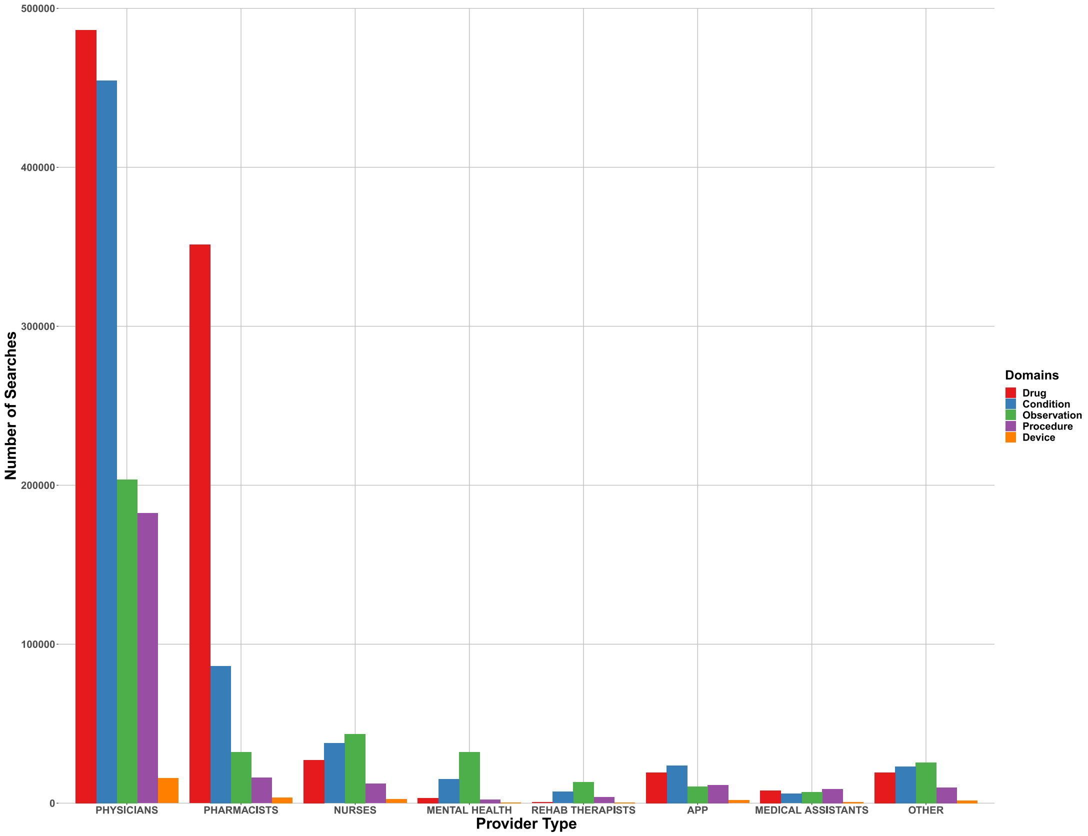

OHDSI= Observational Health Data Sciences and Informatics
APP= Advanced Practice Provider

© 2020 Ruppel H et al. *JAMA Network Open.*

eFigure 3. Network graph: Top 5% of linked searches performed by physicians in internal medicine.

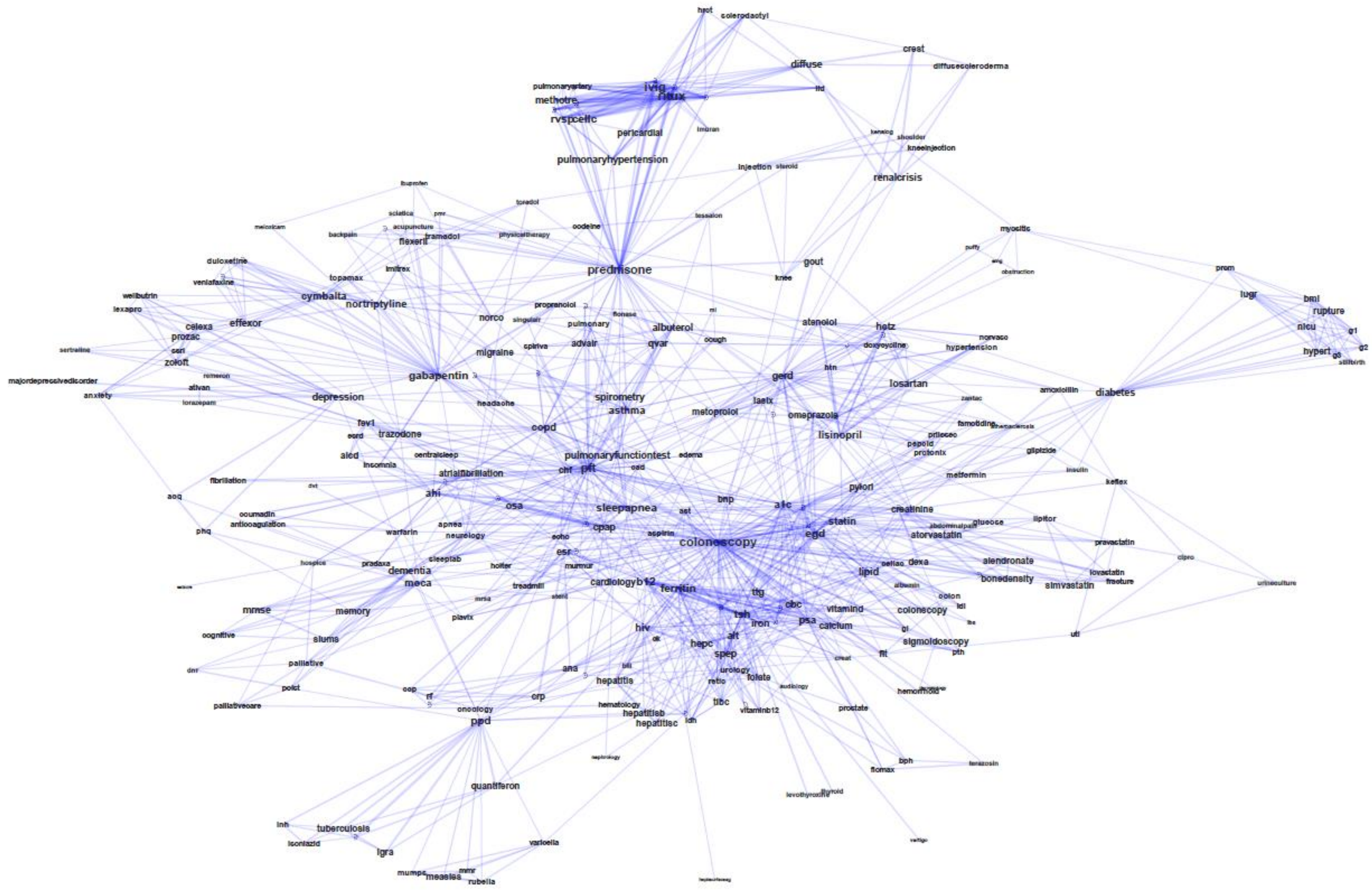

For visual clarity, the network was restricted to the top 5% of most frequently searched pairs of terms. Additionally, terms with fewer than four other terms connected to it were suppressed in this graph. The size of the search term text corresponds to the frequency with which that term was searched.

© 2020 Ruppel H et al. *JAMA Network Open.*
